# The Future of *Carica papaya* Leaf Extract as an Herbal Medicine Product

**DOI:** 10.3390/molecules26226922

**Published:** 2021-11-17

**Authors:** Maywan Hariono, Jeffry Julianus, Ipang Djunarko, Irwan Hidayat, Lintang Adelya, Friska Indayani, Zerlinda Auw, Gabriel Namba, Pandu Hariyono

**Affiliations:** 1 Faculty of Pharmacy, Universitas Sanata Dharma, Campus III, Paingan, Maguwoharjo, Depok, Sleman, Yogyakarta 55282, Indonesia; jeffry@usd.ac.id (J.J.); ipang@usd.ac.id (I.D.); lintangadelya@gmail.com (L.A.); friskadwiindayanii@gmail.com (F.I.); zerlindaclaraa@gmail.com (Z.A.); gabrielnamba21@gmail.com (G.N.); michaelpandu99@gmail.com (P.H.); 2 PT Industri Jamu dan Sido Muncul Tbk., Soekarno Hatta Street Km. 28, Bergas, Klepu, Semarang 50552, Indonesia; hidayat_irwan@sidomuncul.co.id

**Keywords:** *Carica papaya*, leaf, extract, herbal, medicine, future

## Abstract

*Carica papaya* (papaya) leaf extract has been used for a long time in a traditional medicine to treat fever in some infectious diseases such as dengue, malaria, and chikungunya. The development of science and technology has subsequently made it possible to provide evidence that this plant is not only beneficial as an informal medication, but also that it has scientifically proven pharmacological and toxicological activities, which have led to its formal usage in professional health care systems. The development of formulations for use in nutraceuticals and cosmeceuticals has caused this product to be more valuable nowadays. The use of good manufacturing practice (GMP) standards, along with the ease of registering this product facilitated by policies of the national government, will absolutely increase the value of papaya leaf extract as a vital nutraceutical and cosmeceutical products in the near future. In this article, we review the potential of papaya leaf extract to be a high-value commodity in terms of its health effects as well as its industrial benefits.

## 1. Introduction

In tropical and sub-tropical regions, there are abundant flora and fauna living in good climates and circumstances. Flora in particular can constitute valuable sources of various kinds of beneficial products such as dyes, edible tubers, oil crops, furniture, agricultural implements, ornamental plants, pharmaceutical products, rubbers, timbers, and cosmetics [1]. Therefore, the preservation of biodiversity is a compulsory task for the protection of local ecosystems from destruction and to promote healthy conditions for organisms to thrive [2]. There are several ways to promote and to preserve our local biodiversity, including supporting local farms, protecting bees, planting local flowers, fruits, and vegetables, taking shorter showers, respecting local habitats and knowing the sources of products [3]. 

*Carica papaya* (papaw or papaya) is one of the tropical and subtropical trees that is well known for having the entirety of its parts utilized. As a tropical species, this plant grows continuously during winter, although growth slows down and fruit production ceases during the colder months [4]. In Indonesia, the export demands for papaya come from Germany, Hong Kong, Japan, Malaysia, Singapore, Taiwan, and the USA; despite still being high, the export volume decreased from 2009 to 2020 [5]. This could indicate the preservation of this crop becoming less intensive, leading to a reduction in its productivity.

The productivity of papaya has declined, along with the arising of challenges to its fronting, as a result of papaya dieback disease, which could jeopardize its future [6]. One of the dieback diseases is *Erwinia mallotivora*, a phytopathogen bacteria, which plays significant roles in terms of overcoming and limiting the effect of this vulnerable crop [7]. It has 4824 Kbp and the G + C content of the genome detected in papaya showed 52–54% homology to that of reference genomes of other Erwinia species [8]. This information is useful for elucidating the infection mechanism of this disease, based on which the pathway inhibition strategy can be understood. Although all parts of the papaya crop have been widely studied, the fruit and the leaves are two major parts that are used daily for various purposes such as in foods, medicines, pesticides and cosmetics [9].

In foods, the fruit has been advantageously used as a nutritional supplement [10], appetizer [11], and snack [12], whereas as an herbal medicine, the leaves have been utilized in antimicrobe [13], antioxidant [14], antivirus [15,16], haematology disorder treatment [17], and antitumor applications [18]. Although it is minor, the papaya seeds have been studied for their antidiabetic activity [19]. In non-food and non-medicine applications, papaya leaves are also used as a bioherbicides [20], ectoparasite controls [21], and larvicides [22], as well in the control of the onion pest *Spodoptera exigua* [23]. 

As a nutritional supplement, the fruit can be used as a detoxifier and as a metabolism inducer, as well as for rejuvenating the body and maintaining the body’s homeostasis since it is rich in antioxidants, B vitamins, folic acid, pantothenic acid, K and Mg as well as fibre [24]. In particular, B vitamins serve as the co-enzymes in a vast array of metabolism enzymatic reactions. Their cumulative effects are most likely to impact various aspects of brain function, including energy production, DNA/RNA synthesis/repair, genomic and non-genomic methylation, and the synthesis of numerous neurotransmitters as well as signalling molecules. As an appetizer, it was reported that the most common flavour-active compounds in papaya were linalool and benzaldehyde [11]. Therefore, the presence of linalool might be responsible for a different characteristic sweet-flowery flavour in the foods. Another flavour that can induce appetite is benzaldehyde, which can undergo auto-oxidation to per-benzoic acid and can then react with a second molecule of benzaldehyde to become benzoic acid. Upon hydrogenation of benzaldehyde, benzyl alcohol will be formed. It was found that these diverse benzaldehyde products could lead to a range of flavours in papaya fruit [11].

The antibacterial potential of papaya leaves is due to their phenolic compound characteristics, which allows them to react with proteins to form stable water-soluble compounds, thereby killing the bacteria by directly damaging their cell membranes. Flavonoids are a major group of phenolic compounds that are reported to have antiviral, antimicrobial and spasmolytic properties [13]. In diabetes-related applications, papaya seed extract was investigated in terms of its role in increasing glucose transporter 4 (GLUT 4) expression in the skeletal muscle cells of diabetic rats, thereby increasing glucose uptake into cells. With this increased expression of GLUT 4 in muscle cells, it was found that it could be expected to prevent cell atrophy [19]. 

In cosmetics, black hair dyes and face masks can be made from papaya seeds [25] and leaves [26], respectively. In relation to pharmaceutical products, various studies have been conducted to formulate papaya products operating via various routes of administration, including oral [27], topical [28], and transdermal [29]. More specifically, most papaya-based products administered via the oral route were prepared in capsule and tablet dosage forms [30,31], but others, using the liposome delivery system [32] and self-nanoemulsion [33], were also formulated. Various topical dosage forms have been prepared including cream [34], lotion [35], hand sanitizer [36], ointment [37] and emulgel [38].

For example, in cosmetic applications, papaya seeds containing alkaloids, flavonoids, saponins, tannins and triterpenoids were formulated at three different concentrations (2.5, 3.5 and 4.5%) using triethanolamine, glycerol monostearate, tragacanth, stearic acid, cera alba, carnauba wax, ozokerite, coconut fatty acid, methylparaben, solid paraffin and butyl hydroxy toluene. Various tests including a homogeneity test, a consistency test, an effectiveness test and a safety test found that the formula with 4.5% papaya seed extract was good, stable, effective and safe in its use in hair-blackening preparations, and that it met cosmetic requirements [25]. A face mask from papaya leaf powder was formulated using 70% ethanol, rice starch, corn starch, kaolin, benzoic acid, and rose oil by applying the mask on a 2.5 cm area of the upper arm of respondents. A safety test was performed for 24, 48 and 72 h showed that the product did not cause irritation in the 20 respondents [26].

Phytochemical compounds have been identified in papaya leaf, mostly in such classes of flavonoids as apigenin, catechin, deoxyquercetin, hesperitin, isorhamnetin, kaempferol, myricetin, naringenin, protocatechuic acid, quercetin, and rutin. Meanwhile, the fruit is enriched by amino acids, proteins, carbohydrates, fibre, vitamin C, and other nutrients [39]. Interestingly, the all parts of papaya mainly express the white latex, which contains high contents of a proteolytic enzyme called papain, which has been studied in relation to its crucial role in the pathophysiology of many diseases, as well as for drug design, and industrial uses such as in meat tenderizers and pharmaceutical preparations [40].

One of the local products that is formulated as a herbal extract is papaya leaf extract, prepared in capsule dosage form, manufactured by Sido Muncul Industry, located in Semarang, Central Java, Indonesia, as a herbal and pharmaceutical product. In this product, the ingredient is claimed to be a food supplement that helps in weight gain and increasing appetite. In Indonesia, the papaya leaf extract was registered as “jamu”, and it is used more as a dietary supplement (health prevention) than in medications. One of the chemicals causing a bitter taste in papaya leaf extract may come from papain. Papain is a proteolytic enzyme that breaks down proteins into their building block “amino acids”. These amino acids are then easily absorbed by the intestinal membrane, which gives rise to more comfortable abdominal indigestion symptoms such as reduced bloating and loss of appetite [41]. Most likely, people do not enjoy papaya leaf due to its bitter taste. This product is suggested to be suitable for those who want to gain weight by natural means. This product is composed by 500 mg of leaf extract, which is equal to 3 g of its dried leaf, and is indicated to have uses in reducing fever, recovering from dengue infection, malaria, and chikungunya. This product has the advantages of containing various proteins, as well as iron, calcium, vitamins A, B1, and C, and various alkaloids, enzymes, and ribosomal-activating proteins [42].

Good manufacturing practice (GMP) has been applied to standardize the product by conditioning it at a temperature lower than 60 °C in order to maintain the stability of its active ingredients. This product has been registered and licensed by the National Agency of Drug and Food Control. It is contraindicated for pregnant and breast-feeding women. The recommended dose regimen for health promotion is one capsule per person three times daily, which is indicated for those aged 12 and above. Meanwhile, the recommended dose for fever recovery is two capsules per person three times daily. For children 6–12 years old, one capsule per day is sufficient. A sub-chronic toxicity evaluation confirmed the safety of this product for long-term consumption [42].

This article reviews and offers perspectives on some of the most important issues relating to papaya leaf extract, in terms of its applications as a major commodity in the health industry. In the following sections, the extraction process of papaya leaf extract, its various dosage forms, its uses in nutritional supplements and phytochemical substances, its indications, the use of GMP, its product registration, and its safety will be overviewed. References will be made to the indications stated on the label of the papaya leaf extract produced by Sido Muncul, which will be reviewed with a focus on infectious diseases only. Furthermore, the pharmacological and toxicological properties of papaya leaf extract will be discussed, even in terms of its molecular mechanisms, and, on this basis, new indications will be suggested. 

## 2. Extraction

The extraction of papaya leaf is carried out by means of various methods, from traditional maceration, percolation, and Soxhlet approaches to the use of more advanced instruments such as microwaves and ultrasonic cleaners. Table 1 presents the extraction methods used for papaya leaf along with the solvents involved in these methods. The leaves are mostly extracted using a maceration method that employs 96% ethanol as the solvent. The reason for maceration being the most common method could be that it is simpler and less expensive compared to other methods. The leaf has a soft texture; therefore, it is easier for the solvent to penetrate the leaf cells while extracting the phytoconstituent. However, this method has a disadvantage in terms of the equilibrium state between the outside and inside of the cell since the solvent does not move. This causes the extraction to stop, and the residues of filtrate need to be re-macerated using a new solvent [43,44]. On the other hand, 96% ethanol and water are used as two most common solvents. The main reason for choosing these solvents is the fact that they have fewer toxic properties compared to others [45]. Most likely, the flavonoid glycoside as well as alkaloids in a salt form would be easily extracted from the leaf cells due to their suitable polar character in relation to the solvents. Appropriately in light of the above, these two solvents are the most recommended by the National Agency of Drug and Food Control.

## 3. Nutraceuticals/Cosmeceuticals Product

Nutraceutical is a term that is based on the words ‘nutrition’ and ‘pharmaceutical’. In general, nutraceuticals are foods or ingredients that have a significant effect in terms of modifying the workings of the body and maintaining its normal physiological function. Nowadays, nutraceuticals constitute a growing global market and have become an lifestyle-related health promotion trend. The nutraceutical products can be grouped as dietary fibre, prebiotics, probiotics, polyunsaturated fatty acids, antioxidants, and other types of herbal/natural foods. Many metabolic disorders such as obesity, cardiovascular diseases, cancer, osteoporosis, arthritis, diabetes, and cholesterol are controlled via supplementation with nutraceuticals. This is supported by the recent re-orientation of research, which has exhibited a tendency towards focusing on ‘nutraceuticals’ as the one of the most important sectors in the pharmaceutical industry [64].

Cosmeceuticals represent a new category of products that form a hybrid between cosmetics and pharmaceuticals. These products intend to enhance both health and skin beauty. The skincare industry is an ever-increasing part of the healthcare sector, in which cosmeceuticals are formulated from a multitude of ingredients. These developments have caused physicians to consider cosmeceuticals, and subsequently, to recognize and understand their benefits, limitations, and potential adverse effects, which ensures that patients have broad access to these products [65].

The nutraceutical and cosmeceutical products of papaya leaves are mostly manufactured in Asia, especially in India [66]. In this country, there have been at least 58 papaya leaf extract products manufactured in tablet dosage form by diverse pharmaceutical companies. Papaya leaf tablets usually contain 1100 mg of extract per tablet. Other minor products such as capsules, oral drops, and tinctures are produced in US and Indonesia [67,68,69,70]. Various cosmeceutical products are produced in Hungary, particularly skincare products such as toners, cleansers, peeling gels, eye creams, face creams, body polishes, face serums, and skin-clearing masks [71].

## 4. Nutritional and Phytochemical Substances

Nutraceutical and cosmeceutical products are formulated with the consideration of a few factors, in which either nutritional or phytochemical substances play the most essential role in health effects. A number of articles have reported on these aspects [72,73,74]; in general, the nutrients of papaya leaves can be categorized as macromolecules, fibre, minerals, and vitamins [75]. Table 2 summarizes the nutritional substances that have been reported as being present in papaya leaves.

**Figure 1:**
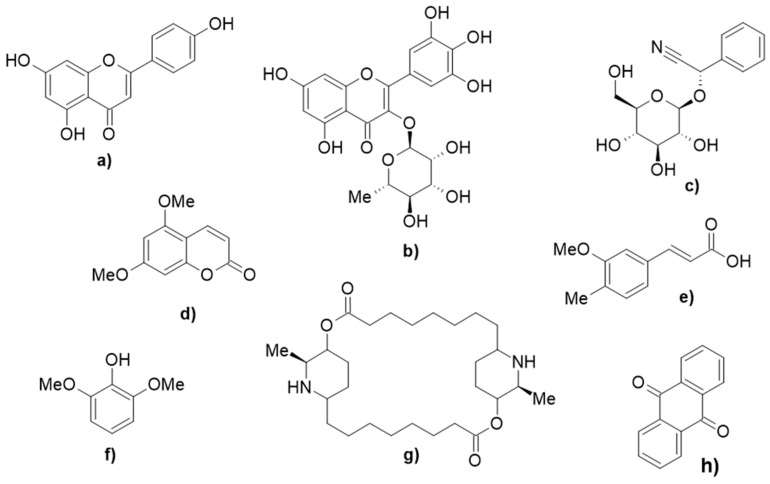
The representative compounds deposited in papaya leaf from each class: (**a**) apigenin, (**b**) myricetin 3-rhanmnoside, (**c**) 2*S*-sambunigrin, (**d**) 5,7-dimethoxycoumarin, (**e**) ferulic acid, (**f**) 2,6-dimethoxyphenol, (**g**) carpaine, and (**h**) anthraquinone [79,80,81,82,83,84,85,86,87,88].

In the literature, the phytochemical compounds in papaya leaf have been summarized and are reported to include: 2*S*-sambuningrin, 5,7-dimethoxycoumarin, anthraquinone, apigenin, caffeic acid, caffeoyl alcohol, catechin, deoxykaempferol, deoxyquercetin, dimethoxyphenol, ferulic acid, kaempferol, *p*-coumaric acid, *p*-coumaric alcohol, protocatechuic acid, *R*-prunasin, carpaine, pseudocarpaine, dehydrocarpaine I, dehydrocarpaine II, carposide, emetine, quercetine 3-(2-rhamnosylrutinoside), kaempferol 3-(2-rhamnosylrutinoside), quercetin 3-rutinoside, myricetin 3-rhamnoside, chlorogenic acid, *E*-3-(4-hydroxy-3-(3,4,5-trimethoxybenzyl)phenyl)acrylic acid, galic acid, and *o*-coumaric acid [79,80,81,82,83,84,85,86,87,88]. Table 3 summarizes and groups the compounds identified in papaya leaves into different classes of natural compounds, whereas Figure 1 depicts the structures of representative compounds from each class that are deposited in papaya leaves. Lists of the compounds identified in papaya pulp and seeds are provided in Tables S1 and S2 (Supplementary Material), respectively [75].

Papaya is not dissimilar to papain (E.C.3.4.22.2), a proteolytic enzyme, which is abundantly (80%) present in latex instead of other existing enzymes such as chymopapain, caricain, acid phosphatase, amylase, chitinase, endo-1,3-*β*-glucanase, glutamine cyclotransferase, lysozyme, peroxidase, and lipase [85]. Papain is a simple cysteine protease enzyme composed of 212 amino acid residue chains with molecular weights of 21,000–23,000 g/mol or 23,406 Dalton. This protease activity is optimal at pH 6.0 to 7.0. Papain consists of a single polypeptide chain with three disulfide bridges and a sulfhydryl group, which are responsible for the activity of the enzyme. By using casein as the substrate, papain shows a low Michaelis–Menten constant (K_m_ = 248.68 ppm) along with a high V_max_ (1.514 ppm casein/min) in its Michaelis–Menten equation, demonstrating its highly active and fast biocatalytic properties [93]. The catalytic site is surrounded by amino acid residues such as GLN19, CYS25, HIS158, and HIS159 [94].

**Figure 2:**
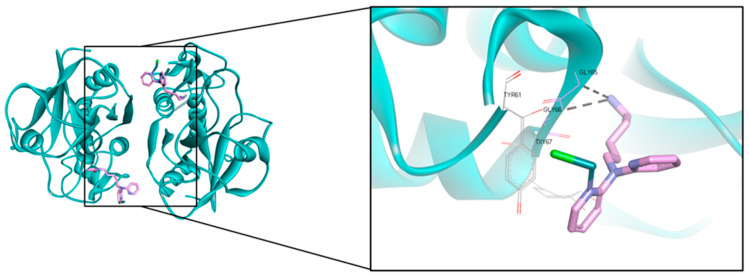
A homodimer of 2.1 Å resolution structure of the complex obtained by aerated overnight conjugation of [(*Z*6-benzene)Ru(1-{5-[*bis*(pyridin-2-yl)]pentyl}pyrrole-2,5-dione)Cl]Cl with papain. The protein is presented in a cyan ribbon and the ligand is in a pink stick model for C, and blue for N.

The latest 3D structures of papain that were deposited in the Protein Data Bank were coded as 6H8T [95]. The resolved structure of the complex obtained by aerated overnight conjugation of [(*Z*6-benzene)Ru(1-{5-[*bis*(pyridin-2-yl)]pentyl}pyrrole-2,5-dione)Cl]Cl with papain is of homodimer type with a resolution of 2.1 Å of (Figure 2). The ligand complexes to the active site and interacts with the cysteine residue (CYS25) by means of the Michael addition of the thiolate to the double bond of the maleimide ring. This leads to hydroxylation to the tyrosine residue while interacting with GLY66 via the H-bond interaction. From this result, it is concluded that due to the modification of the tyrosine residues, this would be a good model in terms of developing a general understanding of the mechanisms and constraints of reactive oxygen species (ROS)-induced damage to proteins.

## 5. Indications

### 5.1. Dengue

In Asia, the use of papaya leaf extract in the treatment of fevers caused by virus infections such as dengue, malaria, and chikungunya is well known. For the treatment of dengue, the use of papaya leaf extract to reduce thrombocytopenia papaya, a condition in which the platelet count is less than 150,000 per µL of blood leaf extract, has been studied. This could be more prevalent because of decreased platelet production and/or their increased destruction. The active ingredients of papaya upregulate the arachidonate 12-lipooxygenase (ALOX 12) and the platelet-activating factor receptor (PTAFR) gene, leading to an increased production of megakaryocytes and their conversion into platelets [96]. A total of four trials, in which 439 subjects were enrolled, were included in the analysis. Of the 439 subjects, data on 377 subjects were available for analysis. From a clinical perspective, it was found that papaya leaf extract increased the platelet count in 377 subjects after the fourth day. However, after 48 h, there was no significant difference between the papaya group and the control group. Interestingly, there was a significant decrease in hospitalization days in the papaya group [16]. It was hypothesized this prevention of blood lysis could have been due to the effects of the flavonoids and other phenolic compounds present in the papaya leaf [97]. The alkaloid carpaine showed anti-thrombocytopenic activity in busulfan-induced thrombocytopenic Wistar rats. In addition, the papain enzyme has been reported to reverse immune-mediated platelet destruction [98,99,100].

However, a systematic review and meta-analysis was performed to study the effects of papaya leaf extract in dengue patients in four different countries including Indonesia, Malaysia, Pakistan, and India. The study found that from a clinical perspective, the platelet count improvement or early discharge was unclear in the absence of more robust indicators of favourable clinical outcome. This led to the claim being made that papaya leaf extract’s ability to reduce thrombocytopenia in dengue patients is insufficient. Therefore, it essential that further well-designed clinical trials are conducted to examine the effects of papaya on platelet counts, plasma leakage, other serious manifestations of dengue, and mortality, with clearly defined outcome measures [101]. Instead of it being hypothesised that the effects of the papaya leaf extract on dengue were due to the reduction in thrombocytopenia, other hypotheses have been made. The presence of the flavonoid quercetin in papaya leaf extract was able to combat the replication of the dengue virus through the inhibition of NS2B/NS3 protease. This protease is important in terms of cleaving polypeptide-constructing structural proteins to produce new virion packages. Therefore, a compound that—either competitively of non-competitively—inhibits this protein could be marked as a dengue antivirus candidate [102,103,104].

**Figure 3:**
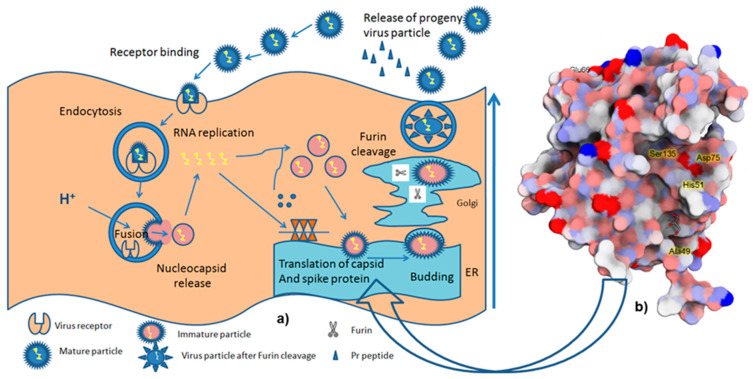
Illustration of (**a**) the life cycle of dengue virus (modified from [108]), and (**b**) the DENV2 NS2B/NS3 protease, retrieved from PDB 2FOM, processing polyprotein cleavages either co-translationally or post-translationally [109].

Figure 3 shows the life cycle of dengue virus. The viral replication cycle is initiated by the infection of flavivirus to the host cells, such as the monocytes, macrophages, and dendritic cells. E protein mediates the penetration of the virus via the endocytosis process. In the endosomal compartment, the virus is acidified, triggering its fusion to the host cell membrane followed by the release of the nucleocapsid and viral RNA into the cytoplasm. The negative strand RNA is formed and serves as a template for further replication, generating positive sense-RNA molecules that provide RNA packaging as well as virus assembly. These coordinates produce the new virions, which allow the maturation of the virus in the Golgi apparatus, and then secrete them through the host secretory pathway. One of the serine proteases of the virus, NS2B-NS3, supported by host-encoded proteases (signalase and furin), processes the translation of the releasing material to generate a polyprotein either co-translationally or post-translationally [105,106]. NS2B-NS3 can be used as an optimal target in dengue drug discovery since it is required for the post-translation of polyprotein as well as the maturation of the virus. Thus, the inhibition of this enzyme is a promising strategy to combat several cases of dengue hemorrhagic fever (DHF) and dengue shock syndrome (DSS) [107].

### 5.2. Malaria

Although malaria is the deadliest tropical disease, it is often neglected by the pharmaceutical industry due its endemic status [110]. Therefore, there are few options in terms of drugs to treat this disease, while resistance is increasing [111]. The drugs to treat malaria are so far categorized into three groups: aryl aminoalcohol compounds (quinine, quinidine, chloroquine, amodiaquine, mefloquine, halofantrine, lumefantrine, piperaquine, and tafenoquine), antifolate compounds (pyrimethamine, proguanil, chlorproguanil, and trimethoprim), and artemisinin compounds (artemisinin, dihydroartemisinin, artemether, and artesunate) [112].

Alternatively, indigenous people consume papaya leaf aqueous extract to reduce fever symptoms caused by malaria. The empirical observations of this medication led to a pre-clinical trial, in which the combination of papaya leaf extract with *Vernonia amygdalina* demonstrated synergistic effects in the amelioration of plasmodium infection in mice. The results showed that the parasite percentage loads between the infected treatment groups and disease control group at day 3 after infection were significantly different (*p* < 0.05). This result maintained its difference until the final experiment, in which all treatment groups showed significant increases in red blood cell (RBC) counts and packed cell volume (PVC), compared to the disease control. In contrast, the white blood cell (WBC) count was reduced, indicating the lowering of the infection status. Moreover, the treatment groups showed a significant elevation of their body weight compared to the disease control group. Meanwhile, the hepatic cells’ histological profile indicates the reduction in its cell damage, thus highlighting that papaya leaf extract is important in the treatment of malaria infection [113].

An in vitro study of papaya leaf extract against *Plasmodium falciparum* (D10 strain) was performed by preparing the extracts in five different solvents. The activities of the five extracts were expressed in IC_50_ values as follows: 16.4 µg/mL (petroleum ether), 12.8 µg/mL (dichloromethane), 2.6 µg/mL (ethyl acetate), 10.8 µg/mL (methanol), and >50.0 µg/mL (water) [114]. This indicates that the most active compound in papaya leaf extract is deposited in either ethyl acetate or methanol. According to the Pfizer guidance on the use of organic solvents, ethyl acetate and methanol are categorized as the preferred solvents; however, the use of methanol could cause serious effects such as acidosis and retinal damage [115]. In a further in vitro study of papaya leaf methanolic extract against *Plasmodium falciparum* (K1 strain), 51% inhibition was observed at a concentration of 4.8 µg/mL. The isolation work revealed some piperidine alkaloids employing (−)-carbamic acid, (+)-methyl carbamate, and (+)-carpaine, along with a (+)-stereoisomer of carpaine and a (+)-derivative of carpaine, which were predicted to be the chemicals responsible for the anti-plasmodium activity. The most potent compound in terms of performance was (+)-carpaine with an IC_50_ of 0.21 µM and a selectivity index of 98, indicating that the potency of this alkaloid is sufficient for it to be an antiparasite of *P. falcifarum* at non-toxic doses. However, in the in vivo murine model, carpaine (daily dose of 10 mg/kg BW intraperitoneally) did not reduce parasitemia until day 10 after infection [116].

### 5.3. Chikungunya

The third mosquito-borne disease, which is traditionally treated with papaya leaf extract, is the chikungunya virus (CHIKV) infection. This arbovirus infection often occurs suddenly without any specific diagnosis, leading to severe clinical manifestations [117]. A study was performed to explore the potency of a methanolic extract of papaya leaf as a CHIKV antiviral agent. This extract showed antiproliferation of infected cells (BHK21) with a CC_50_ of 15.625 µg/mL, whereas the aqueous extract had a CC_50_ of 62.5 µg/mL. Surprisingly, the antiproliferative activity of the papaya leaf extract was better than ribavirin, as the positive control, with a CC_50_ of 125 µg/mL. Two compounds, rutin and carpaine, were isolated from the methanol extract. The CC_50_ values of these compounds against the virus were 125 µg/mL and 15.625 µg/mL, respectively, highlighting the potential effects of this plant as a CHIKV antiviral agent [118].

**Figure 4:**
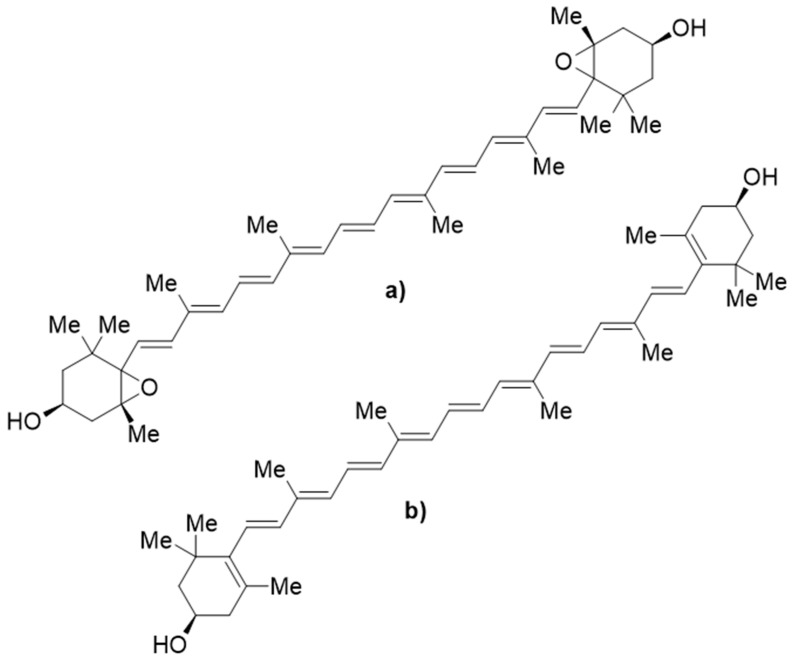
The chemical structures of (**a**) violaxanthin and (**b**) zeaxanthin.

There are few publications on the effect of papaya leaf extract on CHIKV. However, one in silico study was conducted by docking four phytochemicals from papaya leaf, i.e., *p*-coumaric acid, caricaxanthin, violaxanthin, and zeaxanthin. The docking was applied to chikungunya virus glycoprotein (E3-E2-E1) and chikungunya virus non-structural protein2 (nsp2) protease. The result showed that violaxanthin had the best docking score against the glycoprotein E3-E2-E1 via its interaction with ASNB263, but in the docking of nsp2, zeaxanthin showed the best docking score. This work provides insights into the activities of papaya leaf extract active against CHIKV. The chemical structure of violaxanthin and zeaxanthin are presented in Figure 4 [119]; these were not included in the classification of the compounds in papaya leaves in Table 3.

In a further study, papaya leaf extract in the form of silver nanoparticles (AgNPs) was evaluated for its in vitro activity against chikungunya virus (CHIKV), demonstrating a maximum non-toxic dose (MNTD) of 125 μg/mL and a 1/2MNTD 62.5 μg/mL. Compared to the virus control, these toxic doses caused about 39 and 52% of CHIKV inhibition. The treatment using AgNPs showed 14% viability in the infected cells, leading to the conclusion that the AgNPs synthesized from papaya leaf showed antiviral activity against CHIKV when tested on Vero cells [120].

## 6. Good Manufacturing Practice

As with all common pharmaceutical products, the good quality of herbal products must be ensured, not only for the consumers, but also for regulators and manufacturers. The regulations and guidelines in each country can differ due to the political, economic, and cultural policies of each country; therefore, a harmonization of the various standards should be sought out to standardize the good quality of the herbal products.

A published article in 2015 described the major GMP regulations for herbal products implemented in five different regions, i.e., the WHO-GMP, the GMP in China, the current GMP (cGMP) in the United States (US), the Pharmaceutical Inspection Co-operation Scheme (PIC/S) in Singapore, and the GMP in the European Union (EU), to compare them in terms of principles, contents, supervision, and industrial influence. It was found that among the regions, there are major differences in product scope as well as implementation mode. China develops herbal products based on the WHO-GMP, whereas the EU-GMP reflects the PIC/S, and the cGMP of dietary supplements in the US combines multiple GMPs from all regions. For example, without any claims of medicinal activity, herbal products in USA are categorized in dietary supplements, while in the EU-GMP, WHO-GMP or PIC/S, these are regarded as herbal medicinal products. The components of GMP, including personnel, premises and equipment, documentation, production, quality control systems, control and analysis of manufacturing, complaints and product recall, and self-inspection, are almost the same despite occasional differences in expression. In the implementation and supervision of the herbal medicine regulations in different regions and countries, all regions have the same mode of execution, but the agencies, supervising organizations, and sample inspection methods are different. Overall, this study provides consumers, manufacturers, and regulators of herbal products with the means to make decisions regarding the strategy of production of herbal medicine products according to the various GMP standards [121].

In Indonesia, the National Agency of Drug and Food Control implemented a new regulation regarding GMP for herbal medicinal products on June 18th, 2021. This new regulation was constructed to adapt to the recent advancements in science and technology, and to facilitate the ease and supervision of manufacturing, which has gradually been categorized in micro-scale herbal medicine product manufacturing and macro-scale herbal medicine product manufacturing, as a pre-requisite to the releasing of license numbers. A few new points that were highlighted include the following: the amendment of the certification procedure from manual to electronic (e-certificate), the deletion of location agreement requirements by the National Agency of Drug and Food Control, the shortening of service timelines, the prolongation of the GMP for herbal medicine products, and the improvement of GMP for herbal medicine product facilities as well as a reduction in the fee for non-government taxes down to zero (in the IDR currency). The certification of all of the assessed aspects of GMP for herbal medicine products was developed gradually starting from the micro scale and progressing to macro-scale herbal medicine product manufacturing [122].

Sido Muncul, as a national company that specialises in herbal and pharmaceutical products and produces and markets papaya leaf extract, has implemented the GMP standard for herbal medicine products and has been certified accordingly since 2000. Furthermore, it has received the following certifications: ISO 9001:2015 Quality Management Systems, ISO 14001:2015 Environmental Management Systems, ISO 22000:2009 Food Safety Management Systems, Hazard Analysis Critical Control Point (HACPAPAYA), and the Halal Assurance System (SJH). Their products have been certified in the following dosage forms: oral liquid, capsule, soft capsule, pill, poultice, tablet, effervescent powder, semi solid, powder for external medication, and oral powder [123].

## 7. Herbal Medicine Product Registration

In conjunction with GMP, an herbal medicine product should be registered with the National Agency of Drug and Food Control following the consideration of the following factors, and, in so doing, it can be distributed across the globe: (1) the herb must be selected according to the relevant monographs published in its own country or with reference to the WHO monograph. The herbs that have restrictions applied by their countries of origin or by the WHO should be avoided. (2) The chosen part of the plant should be justified in terms of its use. (3) The solvent, extraction technique, the in-process control, optimization and validation must be developed. (4) The herb’s safety, accounts of its traditional usage, and its proposed indications must refer to the solid and reputable studies. (5) A well-planned GMP must be set up; for instance, EU GMP/GLP-approved manufacturing/R&D laboratories are required for EU registrations or USFDA-compliant facilities are required for US registrations. (6) Chemical identifications, including total ash, ash insoluble in hydrochloric acid, heavy metals, loss on drying, extractable matter, residual solvent, etc., must be clearly stated using specified methods such as thin layer chromatography (TLC)/gas chromatography, or other advanced instruments. (7) The marker compound must be justified. (8) The impurity profiles, including insecticides, pesticides, trace metal contents, microbial contamination, and aflatoxins must be clearly stated. (9) Container closure systems and storage conditions must be well defined using stability studies [124].

In Indonesia, an herbal product can be registered in one of three categories, i.e., “jamu”, standardized herbal medicine, and phytomedicine. Jamu is a traditional medicine derived from plants, animals, minerals, and a mixture thereof, which has not been standardized, but rather, is administered medicinally on the basis of experience. It is consumed in various forms including steeping powders, steeping slices, etc. On the other hand, standardized herbal medicines are natural products for which there are standardized results regarding their raw materials, and their uses have been investigated in pre-clinical studies; their efficacy and safety are supported on the basis of in vivo pharmacological and toxicological experiments. Meanwhile, phytomedicines are defined in the same manner as standardized herbal medicines, but they are yet to pass clinical studies [125]. Although it is prepared in capsule form, the papaya leaf extract produced by Sido Muncul is registered as a “jamu” product [42].

## 8. Safety

A full safety study was conducted to evaluate papaya leaf extract in both pre-clinical and clinical settings [126]. Male Wistar rats were given up to 1500 mg/kg of a methanolic papaya leaf extract via gavage, which resulted in no observed mortality [127]. An aqueous papaya leaf extract with a dose of 2000 mg/kg bw led to greater LD_50_ values than those given the methanolic dose in the aforementioned study [128]; meanwhile, there were no mortalities observed when a methanolic papaya leaf extract was administered to Wistar mice in doses of up to 3200 mg/kg [129]. A further study was carried out by giving Wistar rats with a methanolic papaya leaf extract (400 mg/kg bw/d) via gavage for 28 days, it was found that the rats exhibited reduced aspartate aminotransferase activity, enhanced blood urea nitrogen levels, and moderate hyperaemia in the kidney and heart muscles [127]. Another study showed that no extract-related effects were indicated when green papaya leaf extract (up to 2000 mg/kg/day) was administered to Sprague-Dawley rats for 28 days via gavage [130]. Similarly, no adverse effects were shown when Wistar mice were administered a methanolic papaya leaf extract (up to 3200 mg/kg/day) for 60 days [129]. The safety of aqueous papaya leaf extract was evaluated in pregnant Wistar rats via gavage on days 12–18 of gestation with a dose of 60 or 120 mg/kg [131]; deformities were observed in the morphometry of foetuses, while 100% resorption was noted in rats treated with 120 mg/kg of the extract. Other effects of papaya leaf extract on the reproductive system were noted in a study conducted on male Wistar rats [132] given 500 mg/kg bw extract orally for 21 days. This exposure resulted in significant reductions in mean values of sperm count, motility, viability, and serum testosterone concentration, compared to control rats.

Papaya leaf extracts that were mixed with 96% ethanol, followed by partitioning into hexane, ethyl acetate, and water fractions, were evaluated for their cytotoxicity against T47D, a breast cancer cell line, using an MTT assay. The cytotoxicity assay showed that the extract does not interrupt the growth of T47D cells. However, the hexane, ethyl acetate, and water fractions showed a reduced viability of T47D cells with IC_50_ values of 2231.30, 557.33, and 2112.81 µg/mL, respectively. These results showed that the ethanolic extract of papaya leaves and all of its partitions have no potential cytotoxicity in T47D cells due to their high IC_50_ values [56]. 

Recently, a juice and standardized aqueous extract of papaya leaf was reported to be well tolerated by adult humans for short durations (<five days), while one randomised controlled trial reported its safe consumption in children (aged 1–12 years). The most common side effects were minor uncomfortable gastrointestinal feelings. Hepatotoxicity and reproductive toxicity were concerns in relation to long-term use, which was supported by in vivo animal studies. Some unfavourable herb–drug interactions were indicated with metformin, glimepiride, digoxin, ciprofloxacin, and artemisinin. In conclusion, papaya leaf consumption by adults is most likely safe for short-term use, but should be carefully managed when it is given to pregnant women and people with liver impairments. Furthermore, potential herb–drug interactions could occur with oral hypoglycaemic agents, P-glycoprotein substrates, and antibiotics with cation-chelating properties [133]. As previously mentioned, a papaya leaf extract produced by Sido Muncul showed non-sub-chronic toxicity even for long periods of consumption when it was evaluated in an animal study.

## 9. Perspective

In terms of formal medication, traditional medicine is not recognized as the main type of pharmacotherapy due to a lack of evidence-based data. Traditional medicines have been used as alternative medicines by indigenous people due to their difficulties in accessing formal health care facilities or for cultural reasons. Therefore, the production of traditional medicine has historically been conducted via micro-scale home-based industry. This type of industry also seldom produces estimates as to how money has been invested for production as well as the types of marketing undertaken, which has led to unpredictable incomes and non-established businesses.

Papaya is only one of the examples of agro-industrial products that have been developed by the pharmaceutical industry. The plant is easily cultivated in both tropical and semitropical climates, in which all parts of the plant provide numerous benefits, as mentioned in the previous section. In terms of the prospects of papaya as an herbal medicinal product, the leaves are the most widely discussed part of the plant, either in folk preparations or in pharmaceutical dosage forms. In folk medicine, the leaves’ bitter taste and flavour are believed to be a result of ingredients that can cure many illnesses. In the pharmaceutical dosage form, scientists have confirmed the types of those active ingredients as well as providing scientific evidence for their existence through pharmacological and toxicological experiments.

As science and technology has rapidly developed, traditional medicine has begun to attract researchers, especially those who have an interest in conducting evidence-based research on natural products not just in relation to their traditional uses, but also in advance formal medications. The nutritional and phytochemical substances of papaya leaf extract, which could be responsible for its pharmacological activity, have been identified under in silico, in vitro, and pre-clinical in vivo conditions, and even in human clinical studies. The studies were not only carried out at the organism level, but also elucidated the cellular, molecular, and atomic mechanisms relating to the ability of papaya leaf extract to interrupt the pathophysiology of diseases.

Traditionally, papaya leaf extract has been broadly used to treat infectious diseases caused by viruses such as dengue and chikungunya, and also in the treatment of parasites such as malaria. Scientifically, it contains flavonoids, which may exert inhibition to the enzyme NS2B/NS3 protease of the dengue virus that plays a pivotal role in its life cycle. Furthermore, the contents of alkaloid carpaine were reported to potently disrupt the growth of cells infected by *P. falcifarum,* which answers the question as to why papaya leaf extract was traditionally used for malaria fever. In addition, the active phytoconstituents, namely violaxanthin and zeaxanthin, may have an impact on the activity of the chikungunya virus through the inhibition of the E3-E2-E1 glycoprotein and the nsp2 protease enzyme, respectively.

The world was shocked by the outbreak of SARS-Coronavirus-2 (SARS-CoV-2), which, so far, has lasted for almost two years. Although vaccines have been continually developed and distributed in order to urgently bring the pandemic to an end, there is no specific drug to combat the virus [134,135], as was the during outbreaks of dengue as well as chikungunya. In severe SARS-CoV-2 infections, large amounts of inflammation mediators such as tumour necrosis factor-α (TNF-α), macrophages, interleukins (ILs), interferons, and other factors, are present in the lungs, which is widely known as a cytokine storm. This cytokine storm can lead to the cell death, followed by tissue damage and haemorrhages, triggering multiple organ failure. Therefore, by blocking the overproduction of those kinds of inflammation mediators, the severity of infections could be well controlled. Papaya leaf extract was studied in terms of the inhibition of TNF-α rather than IL-6 in the inflammation pathway cascade [136]. A further in vivo study reported that papaya leaf extract was able to alleviate the cytokine storm in a dengue infection mouse model [137]. These two sources of evidence may encourage further experiments on papaya leaf extract as a possible weapon against the COVID-19 pandemic. The protease enzymes and flavonoid contents of other nutrients were also studied in relation to their antioxidant activities [138], as well as their role in T helper-type upregulation and as thrombolytic agents [139], as these biochemical reactions were identified as occurring during COVID-19 pathogenesis, as pointed out in a recent review [140].

The safety of papaya leaf extract was accurately determined, with either in vivo LD_50_ or in vitro LC_50_ values much greater than the effective dose/concentration. However, at certain doses, the use of papaya leaf extract must be carefully handled during pregnancy because the morphometry of foetuses in female rats showed a comparable defect to the negative control. In the male reproductive system, the use of papaya leaf extract should also be carefully managed since this extract was able to reduce the quality and quantity of male Wistar sperm. However, to date, no study has reported toxicity in humans except a that some human subjects experienced unpleasant feelings in their stomach (gastrointestinal disturbance). Furthermore, a drug–herb interaction should also be anticipated when a papaya leaf extract is consumed together with the certain drugs in order to avoid toxicity or reduced efficacy.

In terms of product forms available on the market, papaya leaf extract can be obtained in many dosages and in forms that are acceptable and ready to use. This makes the consumption of papaya leaf extract more practically convenient than drinking the bitter juice extract, and thus, attracts consumers to consume it, not only for medicinal purposes, but also to prevent illness and promote their daily health. Furthermore, the cosmeceutical products obtained from papaya leaf extract enrich the variety of cosmetics intended for use in skincare, which should be a great indicator of the utility of bringing papaya leaf extract into use as a more formal medicine product from its herbal form. It is likely that an increase in guidance related to herbal products in terms of GMP and ISO standards by the National Agency of Drug and Food Control, along with increased ease of product registration, will allow this product to have a strong future in terms of improving the health of consumers as well as bringing economic benefits to the manufacturers.

## 10. Concluding Remarks

A review of *Carica papaya* leaf extract was conducted, highlighting the vast opportunities arising from its potential as a formal herbal medicine product for use in disease prevention, as well as its health-promotion prospects and potential high economic value. A good manufacturing process must be upheld to maintain its quality and sustainability as a nutraceutical and cosmeceutical product. The ease of obtaining governmental support in terms of product registration will boost efforts to develop this product as an advanced pharmaceutical commodity in the near future. To increase the scientific value of *Carica papaya* leaf extract due to its potency as an antiviral agent, especially during the current COVID-19 pandemic, we recommend that research be carried out, starting from in vitro molecular assays and progressing to cellular assays, to evaluate the potential of this extract to inhibit SARS-CoV-2 cell proliferation.

## Figures and Tables

**Table 1:** The extraction method for papaya leaves along with the solvents used.

Extraction Method	Solvents	References
Ultrasonic cleaner	Methanol 96% ethanol	[46] [47]
Hot presser	Water	[48]
Blender	Water	[23,49,50,51]
Maceration	70% ethanol 96% ethanol 70% methanol 80% methanol Water	[25,52] [10,22,27,33,36,53,54,55,56] [57,58] [14,59] [20]
Mixer	cold water, hot water, cold ethanol, 70%	[60,61]
Microwave	methanol, 70% ethanol and water	[62]
Soxhlet	hexane, acetone, 60% ethanol, 40% ethanol and water	[63]

**Table 2:** The nutritional substances reported to be present in papaya leaves [74,76,77,78].

Nutrients	%	Nutrients	%	Nutrients	%
Proteins *	5.8	Phosphorous **	0.221	Vitamin B3 **	0.0003
Lipids *	1.4	Magnesium **	0.032	Vitamin B2 **	0.0001
Carbohydrates *	78.2	Iron **	0.006	Vitamin B1 **	0.0004
Fibre *	13.1	Calcium **	0.366	Vitamin A **	ND
Energy **	348.6 kcal	Vitamin C **	0.031	Beta-carotene **	659.5 IU
Sodium **	ND	Vitamin B9 **	ND		
Potassium **	0.534	Vitamin B6 **	ND		

ND = not determined; * = macronutrient; ** = micronutrient.

**Table 3:** The phytochemical substances reported to be present in papaya leaves.

Class	Compounds	Pharmacological Effects
Flavonoids	apigenin, catechin, kaempferol, deoxykaempferol, deoxyquercetin, protocatechuic acid, galic acid	antioxidant, anti-bacterial, anti-dengue [89]
Flavonoid glycosides	quercetin 3-(2-rhamnosylrutinoside), kaempferol 3-(2-rhamnosylrutinoside), quercetin 3-rutinoside, myricetin 3-rhamnoside	antioxidant [81]
Cyanogenic glycosides	2*S*-sambunigrin, *R*-prunasin	anticancer [90]
Coumarins	5,7-dimethoxycoumarin, *p*-coumaric acid, *o*-coumaric acid, *p*-coumaric alcohol	antioxidant [79]
Quinones	anthraquinone	anti-diabetes [91]
Cinnamic acids	ferulic acid, chlorogenic acid, *E*-3-(4-hydroxy-3-(3,4,5-trimethoxybenzyl)phenyl)acrylic acid	
Phenols	2,6-dimethoxyphenol	antioxidant [92]
Alkaloids	carpaine, pseudocarpaine, dehydrocarpaine I, dehydrocarpaine II, carposide, emetine	antimalarial [83]

## Data Availability

The sample of *Carica papaya* product is available upon request.

## Supplementary Materials

The following are available online. Table S1: The list of macro and micronutrients identified in *Carica papaya* pulp and seeds [75], Table S2: The list of phytochemicals identified in *Carica papaya* pulp and seeds [75].

## References

[1] Kondamudi R., Murthy K.S.R., Pullaiah T. (2009). Euphorbiaceae-a critical review on plant tissue culture. Trop. Subtrop. Agroecosystems.

[2] Romanelli C., Cooper D., Campbell-Lendrum D., Maiero M., Karesh W.B., Hunter D., Golden C.D. (2015). Introduction to the state of knowledge review/Biodiversity and human health linkages: Concepts, determinants, drivers of change and approaches to integration. Connecting Global Priorities: Biodiversity and Human Health: A State of Knowledge Review.

[3] 10 Things You Can Do to Help Biodiversity. http://ecocitizen.tatasteel.com/eco-management/pdf/bio-diversity.pdf.

[4] Allan P. (2000). November. Carica papaya responses under cool subtropical growth conditions. Int. Symp. Trop. Subtrop. Fruits.

[5] Kinding D.P.N. (2021). The financial eligibility of Indonesian calina/ california (*Carica papaya* L.) farm industry. PJSE.

[6] Sekeli R., Hamid M.H., Razak R.A., Wee C.Y., Ong-Abdullah J. (2018). Malaysian *Carica papaya* L. var. Eksotika: Current research strategies fronting challenges. Front. Plant Sci..

[7] Bunawan H., Baharum S.N. (2015). Papaya Dieback in Malaysia: A StepTowards a New Insight of Disease Resistance. Iran. J. Biotechnol..

[8] Mat Amin N., Bunawan H., Redzuan R.A., Jaganath I.B.S. (2011). *Erwinia mallotivora* sp., a new pathogen of papaya (*Carica papaya*) in Peninsular Malaysia. Int. J. Mol. Sci..

[9] Sagadevan P., Selvakumar S., Raghunath M., Megala R., Janarthan P., Vinitha Ebziba C. (2019). Medicinal properties of *Carica papaya* Linn: Review. Madr. J. Nov. Drug. Res..

[10] Ugo N.J., Ade A.R., Joy A.T. (2019). Nutrient Composition of *Carica papaya* Leaves Extracts. J. Food Nutr. Sci. Res..

[11] Rahman A. (2013). Health Benefits, Chemistry and Mechanism of *Carica papaya* a Crowning Glory. Adv. Nat. Sci..

[12] Puji R.P.N., Hidayah B., Rahmawati I., Lestari D.A.Y., Fachrizal A., Novalinda C. (2018). Increasing Multi-Business Awareness through “Prol Papaya” Innovation. IJHSSE.

[13] Baskaran C., Velu S., Kumaran K. (2012). The efficacy of *Carica papaya* leaf extract on some bacterial and a fungal strain by well diffusion method. Asian Pac. J. Trop. Dis..

[14] Nariya A., Jhala D. (2017). Pharmacognostic study of *Carica papaya* leaf extract as inhibitors of reactive oxygen species. Int. Res. J. Pharm..

[15] Sharma N., Mishra K.P., Chanda S., Bhardwaj V., Tanwar H., Ganju L., Kumar B., Singh S.B. (2019). Evaluation of anti-dengue activity of *Carica papaya* aqueous leaf extract and its role in platelet augmentation. Arch. Virol..

[16] Charan J., Saxena D., Goyal J.P., Yasobant S. (2016). Efficacy and safety of *Carica papaya* leaf extract in the dengue: A systematic review and meta-analysis. Int. J. Appl. Basic Med. Res..

[17] Sathyapalan D.T., Padmanabhan A., Moni M., P-Prabhu B., Prasanna P., Balachandran S., Trikkur S.P., Jose S., Edathadathil F., Anilkumar J.O. (2020). Efficacy & safety of *Carica papaya* leaf extract (CPLE) in severe thrombocytopenia (≤30,000/μL) in adult dengue–Results of a pilot study. PLoS ONE.

[18] Mardiyanto N., Dang N.H., Kumagai E., Kondo A., Iwata S., Morimoto C. (2010). Aqueous extract of *Carica papaya* leaves exhibits anti-tumor activity and immunomodulatory effects. J. Ethnopharmacol..

[19] Wulansari D.D., Wulandari D.D., Risthanti R.R., Kirtishanti A. (2019). Ameliorative effect of *Carica papaya* seed extract on diabetic rat model with muscle atrophy. MPI.

[20] Anwar T., Qureshi H., Parveen N., Bashir R., Qaisar U., Munazir M., Yasmin S., Basit Z., Mahmood R.T., Nayyar B.G. (2019). Evaluation of bioherbicidal potential of *Carica papaya* leaves. Braz. J. Biol. Sci..

[21] Azizah L.S., Fasya A.H. (2019). February. Effectiveness of pepaya leaf extract (Carica papaya L.) to control ectoparasite argulus on common carp (Cyprinus carpio). Proceedings of the IOP Conference Series: Earth and Environmental Science.

[22] Kurniawan B., Rapina R., Sukohar A., Nareswari S. (2015). Effectiveness of the pepaya leaf (*Carica papaya* L.) ethanol extract as larvacide for Aedes aegypti Instar III. J. Major..

[23] Permadi M.A., Lubis R.A., Syawaludin S., Pasaribu N.S. (2020). Utilization of papaya leaves (*Carica papaya* L.) to control onion pest *Spodoptera exigua* (lepidoptera: Noctuidae) lepidoptera (noctuidae). Biolink.

[24] Ali A., Devarajan S., Waly M., Essa M.M., Rahman M.S. (2011). Nutritional and medicinal value of papaya (*Carica papaya* L.). Natural Products and Bioactive Compounds in Disease Prevention.

[25] Megantara S., Mustarichie R. (2018). Formulation of black hair dyes in the form of sticks from papaya seed extracts and powder. Int. Res. J. Pharm..

[26] Pratiwi I., Rusita Y.D. (2018). Face mask formulation of papaya leaf extract (*Carica papaya* L.) as anti-acne. JKKT.

[27] Haldar S., Mohapatra S., Singh R., Katiyar C.K. (2020). Isolation and quantification of bioactive Carpaine from *Carica papaya* L. and its commercial formulation by HPTLC densitometry. J. Liq. Chromatogr. Relat. Technol..

[28] Mardiyanto M. (2019). Formulation of ionic-gelation submicron particles loading extract papaya leaves (*Carica papaya* L.) with lactic acid isolates. JSTI.

[29] Viqi K.W. (2020). Formulation of Papaya Seeds Ethanolic Extract Transdermal Patch (*Carica papaya* L.) Using Hydroxypropil Metilcellulose (HPMC) Base. Doctoral Dissertation.

[30] Kumar P.V. (2016). Dengue and drawbacks of marketed *Carica papaya* leaves supplements. IJGP.

[31] Patil S., Ayara P. (2019). *Carica papaya*: Formulation and evaluation of new dosage form design. IJPSR.

[32] Peristiowati Y., Siyoto S., Chusnatayaini A. (2020). The use of sonication method for reducing size of liposomes in papaya leaf extract (*Carica papaya* L.) preparations as a candidate in treatment of cervical cancer. EJMCM.

[33] Nugroho B.H., Citrariana S., Sari I.N., Oktari R.N. (2017). Formulation and evaluation of SNEDDS (Self Nano-emulsifying Drug Delivery System) of papaya leaf extracts (*Carica papaya* L.) as an analgesic. Pharm. Sci. J..

[34] Zukhri S., Andasari S.D., Muchson M. (2018). Formulation and physical quality evaluation of anti-acne cream from papaya leaf extract (*Carica papaya* L.). CERATA JIF.

[35] Shenekar P.N., Ukirade P.S., Salunkhe S.D., Sutar S.T., Magdum C.S., Mohite S.K., Lokapure S.G., Metri S.M. (2014). In vitro evaluation of sun protection factor of fruit extract of *Carica papaya* L. as a lotion formulation. Eur. J. Exp. Biol..

[36] Sari C.M.A., Andriani D., Wahyudi D. (2020). Optimization of HPMC dan Carbopol combination in formulation of papaya seeds ethanolic extract gel (*Carica papaya* L.) and its antibacterial activity against *Escherichia coli*. JIFI.

[37] Prihandiwati E., Sari A.K. (2019). Antibacterial activity evaluation in formulation of papaya leaf hydrocarbon ointment (*Carica papaya* L.) as one of wound healing agent alternatives. JIIS.

[38] Patel N. (2021). Formulation and optimization of synthetic polymer based herbal emulgel for anti-microbial activity. JIAPS.

[39] Hariyono P., Patramurti C., Candrasari D.S., Hariono M. (2021). An integrated virtual screening of compounds from *Carica papaya* leaves against multiple protein targets of SARS-Coronavirus-2. RECHEM.

[40] Olmoss A. (2012). Papain, a plant enzyme of biological importance: A. Am. J. Biochem. Biotechnol..

[41] Vuorinen E., Valtonen S., Hassan N., Mahran R., Habib H., Malakoutikhah M., Kopra K., Härmä H. (2021). Protease Substrate-Independent Universal Assay for Monitoring Digestion of Native Unmodified Proteins. Int. J. Mol. Sci..

[42] Sari Daun Pepaya. https://www.sidomuncul.co.id/en/product/sari_daun_pepaya.html.

[43] Mohammad Azmin S.N.H., Abdul Manan Z., Wan Alwi S.R., Chua L.S., Mustaffa A.A., Yunus N.A. (2016). Herbal processing and extraction technologies. Sep. Purif. Rev..

[44] Hariono M., Rollando R., Karamoy J., Hariyono P., Atmono M., Djohan M., Wiwy W., Nuwarda R., Kurniawan C., Salin N. (2020). Bioguided fractionation of local plants against matrix metalloproteinase9 and its cytotoxicity against breast cancer cell models: In silico and in vitro study. Molecules.

[45] Do Q.D., Angkawijaya A.E., Tran-Nguyen P.L., Huynh L.H., Soetaredjo F.E., Ismadji S., Ju Y.H. (2014). Effect of extraction solvent on total phenol content, total flavonoid content, and antioxidant activity of *Limnophila aromatica*. J. Food Drug Anal..

[46] Fauziah L., Wakidah M. (2019). Extraction of papaya leaves (*Carica papaya* L.) using ultrasonic cleaner. EKSAKTA JSDA.

[47] Utama S.Y.A. (2018). The effect of papaya leaf extract (*Carica papaya* L.) to the bleeding time on mice with thrombocytopenia. IJND.

[48] Kumar M., Sharma P.C., Verma A.K., Sharma A. (2020). Utilization of *Carica papaya* Herbal Leaf Extract for Preparation of a Nutraceutical Functional Beverage. Chem. Sci. Rev. Lett..

[49] Lonkala S., Reddy A.R.N. (2019). Antibacterial activity of *Carica papaya* Leaves and Allium sativum cloves alone and in combination against multiple strains. Pharmacogn. J..

[50] Nurjannah N., Hamidah A., Anggereini E. (2017). Effect of *Carica papaya* Leaf Juice on Hematology of Mice (*Mus musculus*) with Anemia. Biosaintifika.

[51] Hussain S.M., Sohrab M., Al-Mahmood A.K., Shuayb M., Al-Mansur M., Hasan C. (2017). Clinical use of *Carica papaya* leaf extract in chemotherapy induced thrombocytopaenia. Int. J. Clin. Exp. Med..

[52] Pertiwi D., Hafiz I., Salma R. (2019). Antibacterial Activity of Gel of Ethanol Extract of Papaya Leaves (*Carica papaya* L.) againts Propionobacterium acnes. Indones. J. Pharm. Clinical Res..

[53] Sugito S., Suwandi E. (2017). Effectivity of papaya leaf ethanolic extract (*Carica papaya* L.) toward the bacterial growth of *Escherichia coli* using diffusion method. J. Lab. Khatulistiwa..

[54] Gredi J. (2015). Analgesic effectivity of Chitosan-Papaya Leaf Ethanolic Extract Nanoparticle (*Carica papaya* L.) in male white mice (Mus Mucculus). Doctoral Dissertation.

[55] Payangka J., Risma R., Wibowo P. (2019). The influence of papaya leaf extract (*Carica papaya*) toward Aedes agypti INSTAR III mosquito larvae mortality. Med. Health Sci. J..

[56] Yuliani R., Syahdeni F. (2020). Ethanolic extract of papaya leaves (*Carica papaya*) and its fractions have no potential cytotoxicity on T47D Cells. Pharmacon JFI.

[57] Tewari B.B., Subramanian G., Gomathinayagm R. (2014). Antimicrobial properties of *Carica papaya* (Papaya) different leaf extract against *E. coli*, *S. aureus* and *C. albicans*. Am. J. Pharmacol. Pharmacother..

[58] Kamilla L., Tumpuk S., Salim M. (2021). Anti-Inflammatory of papaya leaf extract (*Carica papaya* L.) towards membrane stabilization of red blood cells. JKP.

[59] Hariono M., Hariyono P., Dwiastuti R., Setyani W., Yusuf M., Salin N., Wahab H. (2021). Potential SARS-CoV-2 3CLpro inhibitors from chromene, flavonoid and hydroxamic acid compound based on FRET assay, docking and pharmacophore studies. RECHEM.

[60] Arunkumar S., Muthuselvam M. (2009). Analysis of phytochemical constituents and antimicrobial activities of *Aloe vera* L. against clinical pathogens. World J. Agric. Res..

[61] Liana Y. (2018). Comparative effectiveness of papaya leaf stew (*Carica papaya* L.) with turmeric acid (*Curcuma domestica* val-*Tamarindus indica*) against primary dysmenorrhea. SJM.

[62] Nisa F.Z., Astuti M., Haryana S.M., Murdiati A. (2019). Antioxidant activity and total flavonoid of *Carica papaya* L. leaves with different varieties, maturity and solvent. Agritech.

[63] Singh V., Goyal I., Saini A., Chandra R. (2017). Studying the effect of *Carica papaya* leaf extract on the shelf life of platelets. IJSR.

[64] Das L., Bhaumik E., Raychaudhuri U., Chakraborty R. (2012). Role of nutraceuticals in human health. J. Food Sci. Technol..

[65] Martin K.I., Glaser D.A. (2011). Cosmeceuticals: The new medicine of beauty. Mo. Med..

[66] Medplusmart. https://www.medplusmart.com/compositionProducts/Carica-papaya-leaf-extract-1000-MG/29460.

[67] HerbalGoodness. https://www.herbalgoodnessco.com/.

[68] Sido Muncul. https://www.sidomuncul.co.id/.

[69] Hawaian Herbal. https://hawaiian-Herbal.

[70] New Way Herbs. https://newwayherbs.com/.

[71] INCI Decoder. https://incidecoder.com/.

[72] Saeed F., Arshad M.U., Pasha I., Naz R., Batool R., Khan A.A., Nasir M.A., Shafique B. (2014). Nutritional and phyto-therapeutic potential of papaya (*Carica papaya* L.): An overview. Int. J. Food Prop..

[73] Srivastava A.K., Singh V.K. (2016). *Carica papaya*-a herbal medicine. IJRSB.

[74] Vij T., Prashar Y. (2015). A review on medicinal properties of *Carica papaya* Linn. Asian Pac. J. Trop. Dis..

[75] Santana L.F., Inada A.C., Espirito Santo B.L.S.D., Filiú W.F., Pott A., Alves F.M., Guimarães R.D.C.A., Freitas K.D.C., Hiane P.A. (2019). Nutraceutical potential of *Carica papaya* in metabolic syndrome. Nutrients.

[76] Krishna K.L., Paridhavi M., Patel J.A. (2008). Review on nutritional, medicinal and pharmacological properties of Papaya (*Carica papaya* L.). IJNPR.

[77] Nwofia G.E., Ojimelukwe P., Eji C. (2012). Chemical composition of leaves, fruit pulp and seeds in some *Carica papaya* (L.) morphotypes. Int. J. Med. Aromat. Plants.

[78] Parle M., Gurditta A. (2011). Basketful benefits of papaya. Int. Res. J. Pharm..

[79] Canini A., Alesiani D., D’Arcangelo G., Tagliatesta P. (2007). Gas chromatography–mass spectrometry analysis of phenolic compounds from *Carica papaya* L. leaf. J. Food Compost. Anal..

[80] Akhila S., Vijayalakshmi N.G. (2015). Phytochemical studies on *Carica papaya* leaf juice. Int. J. Pharm. Sci. Res..

[81] Nugroho A., Heryani H., Choi J.S., Park H.J. (2017). Identification and quantification of flavonoids in *Carica papaya* leaf and peroxynitrite-scavenging activity. Asian Pac. J. Trop. Biomed..

[82] Kaur M., Talniya N.C., Sahrawat S., Kumar A., Stashenko E.E. (2019). Ethnomedicinal uses, phytochemistry and pharmacology of *Carica papaya* plant: A compendious review. Mini Rev. Org. Chem..

[83] Julianti T., Oufir M., Hamburger M. (2014). Quantification of the antiplasmodial alkaloid carpaine in papaya (*Carica papaya*) leaves. Planta Med..

[84] Burdick E.M. (1971). Carpaine: An alkaloid of *Carica papaya*: Its chemistry and pharmacology. Econ. Bot..

[85] Azarkan M., El Moussaoui A., Van Wuytswinkel D., Dehon G., Looze Y. (2003). Fractionation and purification of the enzymes stored in the latex of *Carica papaya*. J. Chromatogr. B..

[86] Calvache J.N., Cueto M., Farroni A., de Escalada Pla M., Gerschenson L.N. (2016). Antioxidant characterization of new dietary fiber concentrates from papaya pulp and peel (*Carica papaya* L.). J. Funct. Foods.

[87] Abo K.A., Fred-Jaiyesimi A.A., Jaiyesimi A.E.A. (2008). Ethnobotanical studies of medicinal plants used in the management of diabetes mellitus in South Western Nigeria. J. Ethnopharmacol..

[88] Isolation and Characterization of Secondary Metabolites from Carica Papaya Leaves, Report. https://www.researchgate.net/publication/329828494_Isolation_and_characterization_of_secondary_metabolites_from_carica_papaya_leaves.

[89] Zandi K., Teoh B.T., Sam S.S., Wong P.F., Mustafa M.R., AbuBakar S. (2011). Antiviral activity of four types of bioflavonoid against dengue virus type-2. Virol. J..

[90] Williams D.J., Pun S., Chaliha M., Scheelings P., O’Hare T. (2013). An unusual combination in papaya (*Carica papaya*): The good (glucosinolates) and the bad (cyanogenic glycosides). J. Food Compos. Anal..

[91] Juárez-Rojop I.E., Tovilla-Zárate C.A., Aguilar-Domínguez D.E., Lobato-García C.E., Blé-Castillo J.L., López-Meraz L., Díaz-Zagoya J.C., Bermúdez-Ocaña D.Y. (2014). Phytochemical screening and hypoglycemic activity of *Carica papaya* leaf in streptozotocin-induced diabetic rats. Rev. Bras. Farmacogn..

[92] Zunjar V., Mammen D., Trivedi B.M. (2015). Antioxidant activities and phenolics profiling of different parts of *Carica papaya* by LCMS-MS. Nat. Prod. Res..

[93] Elsson M., Wijanarko A., Hermansyah H., Sahlan M. (2019). Michaelis-menten parameters characterization of commercial papain enzyme “paya”. IOP Conf. Ser. Earth Environ. Sci..

[94] Hansch C., Smith R.N., Rockoff A., Calef D.F., Jow P.Y., Fukunaga J.Y. (1977). Structure-activity relationships in papain and bromelain ligand interactions. Arch. Biochem. Biophys..

[95] Cherrier M.V., Amara P., Talbi B., Salmain M., Fontecilla-Camps J.C. (2018). Crystallographic evidence for unexpected selective tyrosine hydroxylations in an aerated achiral Ru–papain conjugate. Metallomics.

[96] Agarwal A., Vyas S., Agarwal D.P., Pradesh M., Pradesh U. (2016). Therapeutic benefits of *Carica papaya* leaf extracts in dengue fever patients. Sch. J. Appl. Med. Sci..

[97] Ranasinghe P., Ranasinghe P., Abeysekera W.K.M., Premakumara G.S., Perera Y.S., Gurugama P., Gunatilake S.B. (2012). In vitro erythrocyte membrane stabilization properties *of Carica papaya* L. leaf extracts. Pharmacogn. Res..

[98] Sarker M.M.R., Khan F., Mohamed I.N. (2021). Dengue Fever: Therapeutic Potential of *Carica papaya* L. Leaves. Front. Pharmacol..

[99] Zunjar V., Dash R.P., Jivrajani M., Trivedi B., Nivsarkar M. (2016). Antithrombocytopenic activity of carpaine and alkaloidal extract of *Carica papaya* Linn. leaves in busulfan induced thrombocytopenic Wistar rats. J. Ethnopharmacol..

[100] Lavanya B., Maheswaran A., Vimal N., Vignesh K., Yuvarani K., Varsha R. (2018). Extraction and effects of papain obtained from leaves of *Carica papaya*: A remedy to dengue fever. Extraction.

[101] Rajapakse S., de Silva N.L., Weeratunga P., Rodrigo C., Sigera C., Fernando S.D. (2019). *Carica papaya* extract in dengue: A systematic review and meta-analysis. BMC Complement. Altern. Med..

[102] Sulaiman S.N., Hariono M., Salleh H.M., Chong S.L., Yee L.S., Zahari A., Wahab H.A., Derbré S., Awang K. (2019). Chemical constituents from *Endiandra kingiana* (Lauraceae) as potential inhibitors for dengue type 2 NS2B/NS3 serine protease and its molecular docking. Nat. Prod. Commun..

[103] Yap B.K., Lee C.Y., Choi S.B., Kamarulzaman E.E., Hariono M., Wahab H.A., Ranganathan S., Gribskov M., Nakai K., Schönbach C. (2019). In Silico Identification of Novel Inhibitors. Encyclopedia of Bioinformatics and Computational Biology.

[104] Sivasothy Y., Liew S.Y., Othman M.A., Abdul Wahab S.M., Hariono M., Mohd Nawi M.S., Abdul Wahab H., Awang K. (2021). Natural DENV-2 NS2B/NS3 protease inhibitors from *Myristica cinnamomea* King. Trop. Biomed..

[105] Paranjape S.M., Harris E. (2010). Control of dengue virus translation and replication. Dengue Virus.

[106] Rodenhuis-Zybert I.A., Wilschut J., Smit J.M. (2010). Dengue virus life cycle: Viral and host factors modulating infectivity. Cell. Mol. Life Sci..

[107] Niyomrattanakit P., Winoyanuwattikun P., Chanprapaph S., Angsuthanasombat C., Panyim S., Katzenmeier G. (2004). Identification of residues in the dengue virus type 2 NS2B cofactor that are critical for NS3 protease activation. J. Virol..

[108] Idrees S., Ashfaq U.A. (2012). A brief review on dengue molecular virology, diagnosis, treatment and prevalence in Pakistan. Genetic Vaccines Ther..

[109] Erbel P., Schiering N., D’Arcy A., Renatus M., Kroemer M., Lim S.P., Yin Z., Keller T.H., Vasudevan S.G., Hommel U. (2006). Structural basis for the activation of flaviviral NS3 proteases from dengue and West Nile virus. Nat. Struct. Mol. Biol..

[110] Gutman J.R., Lucchi N.W., Cantey P.T., Steinhardt L.C., Samuels A.M., Kamb M.L., Kapella B.K., McElroy P.D., Udhayakumar V., Lindblade K.A. (2020). Malaria and parasitic neglected tropical diseases: Potential syndemics with COVID-19?. Am. J. Trop. Med. Hyg..

[111] Cui L., Mharakurwa S., Ndiaye D., Rathod P.K., Rosenthal P.J. (2015). Antimalarial drug resistance: Literature review and activities and findings of the ICEMR network. Am. J. Trop. Med. Hyg..

[112] Arrow K.J., Panosian C., Gelband H. (2004). Antimalarial drugs and drug resistance. Saving Lives, Buying Time: Economics of Malaria Drugs in an Age of Resistance.

[113] Okpe O., Habila N., Ikwebe J., Upev V.A., Okoduwa S.I., Isaac O.T. (2016). Antimalarial potential of *Carica papaya* and *Vernonia amygdalina* in mice infected with *Plasmodium berghei*. J. Trop. Med..

[114] Melariri P., Campbell W., Etusim P., Smith P. (2012). In vitro antiplasmodial activities of extracts from five plants used singly and in combination against Plasmodium falciparum parasites. J. Med. Plant Res..

[115] Joshi D.R., Adhikari N. (2019). An overview on common organic solvents and their toxicity. J. Pharm. Res. Int..

[116] Julianti T., De Mieri M., Ebrahimi S., Neuburger M., Zimmermann S., Kaiser M., Hamburger M. (2013). Potent antiplasmodial agents in *Carica papaya* L.. Planta Med..

[117] Paixão E.S., Teixeira M.G., Rodrigues L.C. (2018). Zika, chikungunya and dengue: The causes and threats of new and re-emerging arboviral diseases. BMJ Glob. Health.

[118] Tharanatha V. (2017). Screening the Antiviral Activity of *Carica papaya* L. Leaves and Foeniculum Vulgare Fennel Grain Extracts against Chikungunya Virus. Doctoral Dissertation.

[119] Radhakrishnan N., Lam K.W., Norhaizan M.E. (2017). Molecular docking analysis of *Carica papaya* Linn constituents as antiviral agent. Int. Food Res. J..

[120] Kaushik S., Sharma V., Chhikara S., Yadav J.P., Kaushik S. (2019). Anti-chikungunya activity of green synthesized silver nanoparticles using *Carica papaya* leaves in animal cell culture model. Asian J. Pharm. Clin. Res..

[121] He T.T., Ung C.O.L., Hu H., Wang Y.T. (2015). Good manufacturing practice (GMP) regulation of herbal medicine in comparative research: China GMP, cGMP, WHO-GMP, PIC/S and EU-GMP. Eur. J. Integr. Med..

[122] Sosialisasi Peraturan Badan POM Nomor 14 Tahun 2021 Tentang Sertifikasi Cara Pembuatan Obat Tradisional Yang Baik (CPOTB). https://www.pom.go.id/new/view/more/berita/22602/Sosialisasi-Peraturan-Badan-POM-Nomor-14-tahun-2021-tentang-Sertifikasi-Cara-Pembuatan-Obat-Tradisional-yang-Baik--CPOTB--.html.

[123] Sido Muncul. https://www.sidomuncul.co.id/en/certification.html.

[124] Ramadoss M.S.K., Koumaravelou K. (2019). Regulatory compliance of herbal medicines—A review. Int. J. Res. Pharm. Sci..

[125] Diniarti I., Iljanto S. (2017). The strategy of increasing competitive ability in traditional medicine industry (IOT) in Central Java in 2017. JKKI.

[126] Safety Assessment of Carica papaya (papaya)-Derived Ingredients as Used in Cosmetics. https://www.cir-safety.org/sites/default/files/Papaya.pdf.

[127] Nkeiruka U.E., Chinaka N.O. (2013). Anti-fertility effects of *Carica papaya* linn: Methanol leaf extracts in male wistar rats. J. Pharmacol. Toxicol..

[128] Halim S.Z., Abdullah N.R., Afzan A., Rashid B.A., Jantan I., Ismail Z. (2011). Acute toxicity study of *Carica papaya* leaf extract in Sprague Dawley rats. J. Med. Plant Res..

[129] Peristiowati Y., Puspitasari Y. (2017). Acute and subchronic toxicity tests of papaya leaf (*Carica papaya* Linn.) methanol extract on wistar strainwhite mice. J. Appl. Environ. Biol. Sci..

[130] Afzan A., Abdullah N.R., Halim S.Z., Rashid B.A., Semail R.H.R., Abdullah N., Jantan I., Muhammad H., Ismail Z. (2012). Repeated dose 28-days oral toxicity study of *Carica papaya* L. leaf extract in Sprague Dawley rats. Molecules.

[131] Ekong M.B., Akpan M.U., Ekanem T.B., Akpaso M.I. (2011). Morphometric malformations in fetal rats following treatment with aqueous leaf extract of *Carica papaya*. Asian J. Med. Sci..

[132] Akinloye O.O., Morayo O.M. (2010). Evaluation of andrological indices and testicular histology following chronic administration of aqueous extract of *Carica papaya* leaf in Wistar rat. Afr. J. Pharmacy Pharmacol..

[133] Lim X.Y., Chan J.S.W., Japri N., Lee J.C., Tan T.Y.C. (2021). *Carica papaya* L. Leaf: A Systematic Scoping Review on Biological Safety and Herb-Drug Interactions. eCAM.

[134] Hartini Y., Saputra B., Wahono B., Auw Z., Indayani F., Adelya L., Namba G., Hariono M. (2021). Biflavonoid as potential 3-chymotrypsin-like protease (3CLpro) inhibitor of SARS-Coronavirus. RECHEM.

[135] Zubair M.S., Maulana S., Widodo A., Pitopang R., Arba M., Hariono M. (2021). GC-MS, LC-MS/MS, Docking and molecular dynamics approaches to identify potential SARS-CoV-2 3-chymotrypsin-like protease inhibitors from *Zingiber officinale* Roscoe. Molecules.

[136] Tisoncik J.R., Korth M.J., Simmons C.P., Farrar J., Martin T.R., Katze M.G. (2012). Into the eye of the cytokine storm. Microbiol. Mol. Biol. Rev..

[137] Norahmad N.A., Abd Razak M.R.M., Misnan N.M., Jelas N.H.M., Sastu U.R., Muhammad A., Ho T.C.D., Jusoh B., Zolkifli N.A., Thayan R. (2019). Effect of freeze-dried *Carica papaya* leaf juice on inflammatory cytokines production during dengue virus infection in AG129 mice. BMC Complement. Altern. Med..

[138] Ralapanawa U., Alawattegama A.T.M., Gunrathne M., Tennakoon S., Kularatne S.A.M., Jayalath T. (2018). Value of peripheral blood count for dengue severity prediction. BMC Res. Notes.

[139] Bilheiro R.P., Braga A.D., Limborço Filho M., Carvalho-Tavares J., Agero U., das Graças Carvalho M., Sanchez E.F., Salas C.E., Lopes M.T. (2013). The thrombolytic action of a proteolytic fraction (P1G10) from Carica candamarcensis. Thromb. Res..

[140] Potential Herbal-Based Treatment for COVID-19, a Case for Papaya Leaves Extract. https://www.researchgate.net/publication/342053381_Papaya_leaves_extract_a_possible_weapon_against_COVID-19.

