# Supplementary material for: The Future of Carica papaya Leaf Extract as an Herbal Medicine Product
Source: Molecules. 2021 Nov 17;26(22):6922. doi: 10.3390/molecules26226922 (PMC8622926; doi:10.3390/molecules26226922)
Supplement: Supplementary file 1 [file molecules-26-06922-s001.zip › molecules-1438167-supplementary.pdf]

molecules

MDPI

Supplementary Material

The Future of *Carica papaya* Leaf Extract as an Herbal Medicine Product

Maywan Hariono [1,*], Jeffry Julianus [1], Ipang Djunarko [1], Irwan Hidayat [2], Lintang Adelya [1], Friska Indayani [1], Zerlinda Auw [1], Gabriel Namba [1] and Pandu Hariyono [1]

[1] Faculty of Pharmacy, Universitas Sanata Dharma, Campus III, Paingan, Maguwoharjo, Depok, Sleman, Yogyakarta 55282, Indonesia; jeffry@usd.ac.id (J.J.); ipang@usd.ac.id (I.D.); lintangadelya@gmail.com (L.A.); friskadwiindayanii@gmail.com (F.I.); zerlindaclaraa@gmail.com (Z.A.); gabrielnamba21@gmail.com (G.N.); michaelpandu99@gmail.com (P.H.)

[2] PT Industri Jamu dan Sido Muncul Tbk., Soekarno Hatta Street Km. 28, Bergas, Klepu, Semarang 50552, Indonesia; hidayat_irwan@sidomuncul.co.id

* Correspondence: mhariono@usd.ac.id; Tel.: +62-895-0628-6901

Table S1. The list of macro and micronutrients identified in *Carica papaya* pulp and seeds [75].

Component	Pulp (%)	Seeds (%)
Proteins	0.6	2.6
Lipids	0.1	3.1
Carbohydrates	7.2	43.6
Fiber	0.8	2.1
Energy	32.1 kcal	212.7 kcal
Sodium	0.003	ND
Potassium	0.257	0.344
Phosphorous	0.005	0.241
Magnesium	0.01	0.001
Iron	0.0001	0.0002
Calcium	0.024	0.054
Vitamin C	0.062	0.012
Vitamin B9 (folate)	0.038	ND
Vitamin B6	0.0001	ND
Vitamin B3 (niacin)	0.0034	0.0003
Vitamin B2 (riboflavin)	0.00005	0.00005
Vitamin B1 (thiamine)	0.00004	0.00005
Vitamin A	0.328	ND
Betacarotene	888 IU	65.64 IU

Table S2. The list of phytochemicals identified in *Carica papaya* pulp and seeds [75].

Component	Pulp (%)	Seeds (%)
Glutathione peroxidase	ND	ND
Glutathione transferase	ND	ND
Glutathione reductase	ND	ND
Catalase	ND	ND
Glucose-6-phosphate	ND	ND

Total phenols	0.203	0.09
Terpenols	ND	ND
Alkaloids	ND	ND
Flavonoids	ND	ND
Saponins	ND	ND
Benzyl isothiocyanate glucosinolates	ND	ND
Oleic acid	ND	71.30
Palmitic acid	ND	16.16
Linoleic acid	ND	6.06
Stearic acid	ND	4.73
α-Tocopherol	ND	0.0005
δ- Tocopherol	ND	0.0002
β-cryptoxanthine	ND	0.0004
Carotenoids	ND	0.0003

ND = Not determined
